# Family Health Literacy, Family Functioning, and Type 2 Diabetes Self-Management in the Context of Informal Caregiving: An Embedded Mixed-Methods Multiple-Case Study

**DOI:** 10.3390/healthcare14172809

**Published:** 2026-09-01

**Authors:** Filipa Pinheiro, Maria Odete Amaral, Ana Filipa Loureiro

**Affiliations:** 1 Department of Medicine, Hospital Nossa Senhora da Assunção, Local Health Unit of Guarda, 6301-857 Guarda, Portugal; 2 Higher School of Health, Polytechnic University of Viseu, 3500-843 Viseu, Portugal; mamaral@essv.ipv.pt; 3 UICISA:E, University of Coimbra, 3004-011 Coimbra, Portugal; 4 A Serrana Family Health Unit, Local Health Unit of Guarda, 6301-857 Guarda, Portugal; filipa.lou83@gmail.com

**Keywords:** diabetes mellitus, type 2, health literacy, family relations, self-management, family nursing, primary health care, case study

## Abstract

**Background/Objectives**: Type 2 diabetes is a prevalent chronic disease that requires continuous self-management and adaptation by both individuals and their families. Health literacy is recognised as an important determinant of chronic disease management, influencing the ability to access, understand, and use health information. This study aimed to explore how family health literacy and family functioning were related to type 2 diabetes self-management across four family cases in the context of informal caregiving and to describe the changes observed following tailored family nursing interventions. **Methods**: An embedded mixed-methods multiple-case study was conducted with four families receiving care in a Portuguese primary healthcare unit. Qualitative data were obtained through structured family interviews guided by the Dynamic Model of Family Assessment and Intervention. Quantitative data were collected using family assessment instruments and the Diabetes Self-Care Activities Scale. Each family was analysed as an individual case, followed by cross-case comparison and integration of qualitative and quantitative findings. **Results**: Across the four cases, the ability to understand and translate diabetes-related information into family routines varied and was associated with differences in self-management practices. Within-case comparisons identified case-specific changes in family communication, mutual support, selected family nursing diagnoses, and some diabetes self-care behaviours. Medication adherence and blood glucose monitoring were more consistently maintained than dietary management, physical activity, and foot care. **Conclusions**: Family health literacy appeared to operate as a relational and context-dependent resource associated with how diabetes-related recommendations were negotiated and incorporated into daily family routines. Tailored family nursing interventions were followed by case-specific changes; however, the study design does not allow causal attribution.

## 1. Introduction

Type 2 diabetes (T2D) is one of the most prevalent chronic diseases worldwide and represents a major global public health challenge. Effective disease management requires continuous self-care behaviours, including adherence to medication, healthy eating, regular physical activity, and blood glucose monitoring. However, maintaining these behaviours remains challenging for many individuals, particularly older adults living with chronic conditions [1,2].

Health literacy has been increasingly recognised as a key determinant of successful diabetes self-management. Individuals with higher levels of health literacy are generally better able to access, understand, and apply health information, resulting in improved self-care practices and health outcomes. Unlike individual health literacy, which focuses primarily on an individual’s capacity to access, understand, appraise, and use health information, family health literacy concerns how health information is distributed, discussed, negotiated, and translated into action within family relationships. In chronic disease management, health-related knowledge may therefore become actionable not only through individual understanding but also through shared decision-making, communication, role distribution, and the mobilisation of family resources. Conversely, limited health literacy has been associated with poorer disease control, lower treatment adherence, and an increased risk of complications [3,4,5,6].

Diabetes management does not occur in isolation. Family members often play a crucial role in supporting self-management behaviours, influencing health-related decisions, and facilitating adaptation to chronic illness. In the context of this study, informal caregiving refers to unpaid support provided by family members involved in the day-to-day management of diabetes, including diabetes-related support and shared decision-making. Within the family context, health literacy extends beyond the individual ability to access and understand health information. It encompasses the collective capacity of family members to interpret health recommendations, share knowledge, negotiate decisions, organise daily routines, and mobilise formal and informal resources. Family health literacy may therefore function as a distributed relational resource that influences how diabetes-related recommendations are translated into everyday self-management practices. Family-centred nursing interventions may contribute to strengthening family health literacy, enhancing family functioning, and promoting more effective T2D self-management [7,8,9,10,11].

In the present study, family health literacy was conceptualised as a relational and distributed capacity rather than exclusively as an individual attribute. It refers to the family’s collective ability to access, understand, discuss, clarify, negotiate, and translate health-related information into shared decisions and everyday practices. This construct was distinguished analytically from family functioning, which refers to relational processes such as communication, role distribution, adaptability, and collaborative problem-solving; from family support, referring to practical and emotional assistance provided among family members; and from diabetes knowledge, referring to the understanding of disease-related information. Because no validated family health literacy instrument was used, family health literacy was explored through qualitative family narratives and contextualised family assessment rather than quantified as an independent outcome.

Although health literacy and family support have independently been associated with diabetes self-management, less is known about how diabetes-related information is collectively interpreted, negotiated, and translated into everyday family routines. Evidence is also limited regarding how structured family nursing assessment and intervention may address these relational processes, particularly among older adults with T2D receiving care in primary healthcare [4,10,11]. The present study therefore addresses this gap by examining family health literacy and family functioning across individual family cases and by integrating qualitative family narratives with quantitative family and diabetes self-management assessments.

The Dynamic Model of Family Assessment and Intervention (MDAIF) provides an operational framework for examining these processes by considering the structural, developmental, and functional dimensions of the family. Its assessment–diagnosis–intervention–evaluation sequence may support the identification of family needs, strengths, and health gains sensitive to family nursing care [7,8,12].

Therefore, this study aimed to explore how family health literacy and family functioning were related to T2D self-management across four family cases in the context of informal caregiving and to describe the qualitative and quantitative changes observed following tailored family nursing interventions.

## 2. Materials and Methods

### 2.1. Study Design

This study adopted an embedded mixed-methods multiple-case study design [13]. The family constituted the primary unit of analysis, while qualitative and quantitative data were embedded within each family case. The qualitative component explored family experiences, interactions, and interpretations of diabetes-related information, whereas the quantitative component characterised family functioning, social context, and diabetes self-management. The two components were first analysed within each case and subsequently integrated through case summaries, joint displays, and cross-case comparison.

The study was conducted in a primary healthcare unit within a Local Health Unit of Guarda. Ethical approval was obtained from the Health Ethics Committee of the Research Unit of the Local Health Unit of Guarda on 15 November 2024, before data collection commenced. All participants provided written informed consent prior to participation. Confidentiality, anonymity, privacy, and voluntary participation were ensured throughout the study.

### 2.2. Case Selection, Participants and Unit of Analysis

Families were purposively selected according to predefined eligibility criteria and to provide variation in family structure, available support, family functioning, and diabetes self-management needs. The selection followed a replication logic consistent with multiple case study methodology, whereby similarities and differences across cases were examined to identify recurring patterns as well as case-specific characteristics. The purpose of selecting four cases was therefore analytical and contextual rather than statistical, enabling detailed within-case analysis followed by cross-case comparison [13].

Following application of the predefined eligibility criteria, 47 families were identified as eligible for the study. From this pool, four families were purposively selected by the nurse-researcher, considering the predefined variation criteria described above. All four selected families were invited to participate and accepted the invitation, with no refusals or subsequent withdrawals.

Families were eligible if they included at least one person aged 65 years or older diagnosed with T2D and receiving care at the participating primary healthcare unit, and if at least one family member was actively involved in the person’s day-to-day diabetes management. Family members were eligible to participate when they were involved in diabetes-related support or decision-making and provided informed consent.

The family was defined as the primary unit of analysis. Individual family members, interview narratives, family assessment measures, family nursing diagnoses, and diabetes self-care indicators constituted embedded sources of evidence within each case.

### 2.3. Data Collection

Data collection occurred at two main time points: an initial family assessment and a follow-up assessment after implementation of the family nursing intervention plan.

Family interviews were conducted during nursing consultations in a private consultation room. Interviews were audio-recorded with participants’ consent, transcribed verbatim, and reviewed to ensure accuracy and completeness. Field notes were completed after each consultation to document contextual observations, family interactions, and researcher reflections.

#### 2.3.1. Qualitative Component

Qualitative data were obtained through structured family interviews guided by the operational matrix of the MDAIF [12]. Interviews explored how families understood diabetes-related information, organised self-management routines, distributed responsibilities, made health-related decisions, and experienced the family nursing intervention.

#### 2.3.2. Quantitative Component

Quantitative data were collected using the Family APGAR [14], FACES II [15], Adapted Graffar Scale [16], Holmes and Rahe Social Readjustment Rating Scale [17], and the Portuguese version of the Diabetes Self-Care Activities Scale [18]. These instruments were selected to characterise family functioning, social context, stress exposure, and diabetes self-management behaviours. The version and language used for each instrument were those validated or adapted for the Portuguese context, where applicable.

The Family APGAR, FACES II, Adapted Graffar Scale, and Holmes and Rahe Social Readjustment Rating Scale were administered at baseline for family characterisation and to support the structured family assessment and family nursing diagnostic process; they were not used as pre–post outcome measures. The Diabetes Self-Care Activities Scale was used to assess diabetes self-management behaviours at baseline and follow-up, allowing within-case comparison over the intervention period. Given the small number of cases, quantitative findings were analysed descriptively and were not subjected to inferential statistical testing.

### 2.4. Family Nursing Intervention

Following the initial assessment, an individualised family nursing intervention plan was collaboratively developed with each participating family. The intervention process followed the assessment–diagnosis–intervention–evaluation sequence proposed by the MDAIF [12] and was tailored according to the needs, priorities, strengths, and available resources identified within each family. Therefore, the interventions focused on the identified family nursing diagnoses and included tailored health education, therapeutic questioning, clarification of beliefs, reinforcement of family strengths, negotiation of achievable self-management goals, promotion of family communication, and mobilisation of formal and informal support resources. Systemic family nursing techniques were selected according to the needs of each family and included linear, circular, strategic, reflective, and scaling questions, therapeutic metaphors and rituals, positive reinforcement, and encouragement of family members to share their perspectives and support one another.

The number, duration, frequency, mode of delivery, and outcomes of the intervention contacts were documented for each family case. Each consultation lasted approximately 45 min. The number and frequency of contacts varied according to the needs of each family. The follow-up assessment was conducted approximately three months after the baseline assessment, following completion of the planned family nursing intervention period. All family nursing interventions were conducted by the same family nurse who performed the initial and follow-up assessments and participant interviews.

### 2.5. Data Analysis

#### 2.5.1. Qualitative Analysis

Qualitative data were analysed using codebook thematic analysis, following the methodological principles proposed by Braun and Clarke [19]. An initial coding framework was informed by the study objectives and by the structural, developmental, and functional dimensions of the MDAIF, while allowing additional inductive codes to emerge from the family narratives.

Researcher reflexivity was considered throughout the qualitative analysis. The researchers acknowledged that the professional role of the family nurse and her prior knowledge of the participating families could influence data collection and interpretation. To reduce the influence of individual interpretations, three researchers independently reviewed the interview transcripts, discussed coding decisions, refined the coding framework through an iterative process, and collaboratively developed the final themes.

#### 2.5.2. Quantitative Analysis

Quantitative data were summarised descriptively for each family case. Baseline scores from the Family APGAR, FACES II, Adapted Graffar Scale, and Holmes and Rahe Social Readjustment Rating Scale were used for family characterisation. Diabetes Self-Care Activities Scale scores obtained at baseline and follow-up were compared within each case to identify the direction and magnitude of observed changes in diabetes self-management behaviours. Inferential statistical analyses were not performed because the study was designed to support case-level interpretation rather than population-level estimation.

#### 2.5.3. Integration of Qualitative and Quantitative Findings

Qualitative and quantitative data were first analysed separately within each family case and subsequently integrated through case summaries and joint displays. Integration focused on identifying convergence, complementarity, and divergence between family narratives, family assessment measures, diabetes self-care scores, family nursing diagnoses, and observed outcomes.

Following the within-case analyses, a cross-case comparison was undertaken to identify recurring patterns as well as case-specific differences across the four participating families.

## 3. Results

The four family cases differed in family structure, socioeconomic conditions, available support, duration of diabetes, and self-management needs. Table 1 summarises the demographic, clinical, family, and social characteristics of the participating families.

The initial family assessment identified distinct combinations of strengths and needs across the four cases. Although all families demonstrated some capacity to support diabetes management, differences were observed in communication, distribution of responsibilities, understanding of health recommendations, and integration of self-care behaviours into daily routines.

Family nursing diagnoses were established through the integration of interview data, family assessment instruments, clinical observations, and diabetes self-care indicators. Based on these findings, tailored family nursing intervention plans were collaboratively developed according to the identified needs, strengths, preferences, and available resources of each family. Table 2 presents the family nursing intervention process across the four family cases, including the priority nursing diagnoses and care foci, intervention objectives, tailored family nursing interventions, and observed outcomes.

The case-specific outcomes presented in Table 2 were complemented by the quantitative results from the Diabetes Self-Care Activities Scale, expressed as the number of days per week on which each self-care behaviour was reported. Between the initial and follow-up assessments, selected case-specific changes were observed. In Family 2, fruit and vegetable consumption increased from four to five days per week, while bread consumption with main meals decreased from seven to four days per week; in Family 3, bread consumption decreased from seven to five days per week. Family 2 also reported a reduction in the consumption of more than one alcoholic drink with main meals from seven to four days per week, while Family 1 reported a reduction in adding sugar to beverages from seven to five days per week. Specific physical activity increased from zero to two days per week in Family 1 and from zero to three days per week in Family 2. Foot inspection increased from zero days per week at baseline to four, five, and seven days per week at follow-up in Families 1, 3, and 4, respectively. In contrast, blood glucose monitoring, medication adherence, and smoking behaviour remained stable where adequate self-care practices were already established at baseline. Overall, these quantitative findings showed that changes varied across families and self-care domains, complementing the patterns identified in the qualitative case analysis.

Within-case comparisons identified changes between the initial and follow-up assessments in selected family nursing diagnoses, communication processes, mutual support, and diabetes self-care activities. The case-level findings showed different patterns across families. Family 1 showed changes in diabetes self-management behaviours and family involvement in diabetes care. Family 2 showed changes in selected diabetes self-management behaviours, whereas the diagnosis of non-maintained conjugal satisfaction remained unchanged. Family 3 maintained effective family functioning and showed changes in selected diabetes self-management behaviours. Family 4 showed changes in conjugal satisfaction and family process, together with changes in selected self-management behaviours and family support. These findings indicate heterogeneity in the patterns observed across cases rather than a uniform response to the intervention.

### 3.1. Family Health Literacy as a Relational Resource

Across the four family cases, family health literacy was expressed not only through the ability of the person living with T2D to understand health information but also through the collective capacity of family members to discuss recommendations, clarify doubts, organise daily routines, and support health-related decision-making. Families demonstrating greater capacity to translate diabetes-related information into concrete daily practices appeared to adopt more consistent self-management behaviours, whereas families requiring greater support received tailored family nursing interventions to strengthen family health literacy and its application in everyday life.

For example, one spouse described how diabetes recommendations were discussed and adapted within the family before being incorporated into daily life: “*We usually talk about what the nurse told us, and then we decide together how to organise meals and medication so that it works for both of us*” (Family 1, spouse). Similarly, one participant living with T2D emphasised that family support made it easier to understand and apply health recommendations: “*If I have any doubts, I ask my husband first because he helps me remember what I should do*” (Family 3, person living with T2D).

### 3.2. Family Functioning and Support for Diabetes Self-Management

Family functioning was related to how diabetes self-management responsibilities were shared and sustained over time. Families characterised by more effective communication, collaborative problem-solving, and mutual emotional support demonstrated greater capacity to integrate diabetes management into everyday life. In contrast, families presenting communication or relationship difficulties required more intensive family nursing interventions to strengthen adaptive family processes and support diabetes self-management.

This collaborative approach was reflected in participants’ narratives. One participant explained: “*We try to solve things together because diabetes is not only his problem; it affects all of us*” (Family 1, spouse). Another family member highlighted the importance of communication in maintaining self-management behaviours: “*When we talk about it openly, it becomes easier to keep the routine and remind each other of what needs to be done*” (Family 3, spouse).

### 3.3. Family Nursing Interventions and Observed Changes

Following the tailored family nursing interventions, case-specific changes were observed in communication, shared responsibility, family support and diabetes self-management behaviours. The direction and extent of these changes varied across the four family cases. These observations should not be interpreted as evidence of a causal effect of the intervention, given the multiple-case design, absence of a comparison group and short follow-up period. During the intervention period, families reported greater recognition of their existing strengths and described practical strategies for incorporating diabetes management advice into their daily routines.

Qualitative narratives and quantitative indicators provided complementary evidence of changes in diabetes self-management behaviours across the four family cases. Changes in family functioning, however, varied according to the priority family nursing diagnoses identified during the initial assessment. Families presenting difficulties in family functioning demonstrated different patterns of change following the intervention, whereas families with more effective baseline functioning primarily maintained existing adaptive processes.

Participants described becoming more aware of the shared responsibility for diabetes management and reported greater organisation and collaboration in their daily routines. As one participant explained, “*After the consultations, we became more aware that managing diabetes is something we need to do together as a family*” (Family 1, spouse). Similarly, another spouse stated, “*The consultations helped us organise ourselves better and support each other in managing diabetes every day*” (Family 3, person living with T2D). Changes in family communication were also reported, as reflected in the following statement: “*Now we discuss things more calmly and try to help each other instead of arguing about what should be done*” (Family 4, person living with T2D).

### 3.4. Persistent Challenges in Diabetes Self-Management

Medication management and blood glucose monitoring were generally more consistently maintained across the participating families. In contrast, dietary management, physical activity, and foot care remained the most challenging components of diabetes self-management because they required sustained behavioural changes, reorganisation of family routines, and the active involvement of more than one family member.

Although case-specific changes were observed during the intervention period, these domains continued to represent important challenges across the family cases, highlighting the need for ongoing family-centred support and reinforcement of family health literacy within everyday family life. Participants acknowledged that maintaining behavioural changes over time remained difficult despite increased awareness and family support. One participant reflected: “*We know what we should do, but it’s not always easy to keep those habits every day.*” (Family 2, person living with T2D) Another participant similarly acknowledged: “*The consultations helped us get started, but keeping these changes over time is the hardest part.*” (Family 3, spouse).

## 4. Discussion

This study explored how family health literacy and family functioning were related to T2D self-management across four family cases receiving family-centred nursing care in a primary healthcare setting. By integrating qualitative family narratives with family assessment measures, family nursing diagnoses, and diabetes self-care indicators, the study provided a comprehensive understanding of how diabetes management is negotiated and enacted within the family context. The findings suggest that tailored family health nursing interventions were followed by case-specific changes in family processes and selected diabetes self-care behaviours. Across the four cases, these changes were observed alongside greater engagement of the person with diabetes and the family as a caregiving unit, as well as changes in family health literacy, family functioning, and the integration of diabetes self-management recommendations into everyday family life. However, given the multiple-case design, absence of a comparison group, short follow-up period, and potential influence of self-report and repeated contact, these changes cannot be causally attributed to the intervention.

The observed patterns across the sequence of family health nursing interventions suggest that family health literacy may have operated as a family and relational resource rather than exclusively as an individual competence. The ability to understand diabetes-related information alone appeared insufficient when families were unable to negotiate responsibilities, reorganise routines, communicate effectively, or translate recommendations into sustainable daily practices. Across the four cases, families that collectively discussed health information, clarified doubts, and shared responsibilities demonstrated greater capacity to incorporate diabetes management into everyday family life. These findings extend previous evidence linking health literacy with diabetes self-management by suggesting that literacy is not merely an individual cognitive skill but a relational process shaped by family interactions and shared decision-making [20,21,22,23].

This possible relational pattern of family health literacy is also consistent with emerging perspectives that conceptualise health literacy as a distributed family resource rather than an isolated individual competence [3,4]. Diabetes management required not only access to information but also the family’s ability to coordinate action, communicate effectively, mobilise formal and informal support, and adapt recommendations to everyday circumstances. Consequently, family health literacy may be understood as a distributed relational resource influencing how health information is interpreted, negotiated, and translated into daily practice. This interpretation is conceptually aligned with the Community-for-Care framework proposed by Afonso et al., in which care literacy emerges through the interaction between knowledge, relational support, community resources, and coordinated action. Although developed in the context of informal post-caregivers, that framework reinforces the idea that health-related knowledge becomes meaningful only when integrated into family relationships, available resources, and collaborative action, supporting the interpretation advanced in the present study [24].

Consistent with this relational perspective, the findings further highlight the importance of family functioning in chronic disease management. Diabetes self-management extended beyond individual behaviours and depended on communication patterns, role negotiation, emotional support, and collaborative problem-solving within the family. Families characterised by stronger communication and greater mutual support appeared better able to adapt diabetes recommendations to their daily routines and sustain self-management behaviours over time. These observations reinforce the principles of Family Health Nursing, which recognise the family as the unit of care and emphasise the reciprocal relationship between individual and family health. Similar findings have been reported in previous studies demonstrating that active family involvement contributes positively to treatment adherence, self-care behaviours, and adaptation to chronic illness [25,26,27].

The structured use of the MDAIF provided an operational framework that connected multidimensional family assessment, family nursing diagnoses, negotiated interventions, and outcome reassessment. Rather than focusing exclusively on the transmission of health information, the intervention process addressed communication patterns, family roles, relationships, strengths, beliefs, and available support resources. This assessment–diagnosis–intervention–evaluation sequence is consistent with the operational application of the MDAIF described by Afonso and Amaral, who demonstrated that systematic family case analysis supports clinical reasoning, collaborative decision-making, and the translation of family nursing theory into clinical practice. Although their study was conducted within a family nursing educational context, the present findings suggest that the same structured reasoning process may support family-centred clinical practice in primary healthcare [28].

The changes observed during the intervention period may be consistent with the potential value of sequential and tailored family nursing contacts; however, the study design does not allow these changes to be causally attributed to the intervention. The opportunity to revisit information, clarify misunderstandings, negotiate achievable goals, reinforce family strengths, and adapt recommendations to each family’s context coincided with case-specific changes in the incorporation of diabetes management into everyday routines. These observations are consistent with previous research describing the potential relevance of individualised and family-centred approaches to behavioural adaptation and family resources in chronic disease management [25]. Accordingly, the observed case-specific changes should be interpreted as patterns occurring during the intervention period rather than as evidence of intervention effectiveness. Changes in family nursing diagnoses should therefore be interpreted within the context of each case and the specific intervention implemented, rather than as uniform outcomes across all families. Importantly, the persistence of impaired marital satisfaction in one family illustrates that family change is not necessarily linear and that some relational needs may require longer-term or more specialised interventions. These observations are consistent with the findings of Afonso et al., who reported that a structured telephone intervention combining psychoeducation and emotional support enabled family caregivers to recognise their own needs, mobilise personal strengths, develop coping strategies, and sustain hope. Although conducted in a different clinical context, the findings of both studies highlight the potential relevance of tailored and sequential nursing interventions to the integration of knowledge, coping strategies, and family resources into everyday life [29].

Despite the changes observed across several domains, dietary management, physical activity, and foot care remained the most challenging aspects of diabetes self-management. Unlike medication adherence and blood glucose monitoring, these behaviours required sustained modifications of family routines, food preferences, time allocation, financial resources, physical capacity, and shared household practices. These findings suggest that knowledge alone is insufficient to achieve consistent behavioural change and reinforce the importance of addressing contextual and relational determinants of self-management. Similar challenges have been consistently reported in previous studies investigating chronic disease self-management [27,30].

The findings have important implications for Family Health Nursing and primary healthcare practice. Family nurses should assess not only whether people living with T2D understand health information but also how that information is discussed, negotiated, and implemented within the family. Assessment should include family roles, communication patterns, decision-making processes, available formal and informal resources, and the feasibility of incorporating diabetes recommendations into everyday family routines. This broader assessment may inform the development of genuinely family-centred interventions that address family health literacy and family functioning while supporting sustainable diabetes self-management.

Several limitations should be considered when interpreting the findings. First, the purposive selection of four families from a single primary healthcare unit limits the diversity of contexts represented, may restrict the analytic transferability of the findings, and may have introduced selection bias. Second, the absence of a comparison group, the relatively short follow-up period, and the self-reported nature of some outcomes preclude causal attribution of the observed changes to the intervention. Third, the same family nurse was involved in assessment, intervention, and outcome evaluation, potentially introducing interviewer and observer bias and influencing participants’ responses. Fourth, a Hawthorne effect cannot be excluded, as participants may have modified their behaviour in response to repeated contact and increased attention during the intervention period. Finally, family health literacy was explored qualitatively and contextually rather than measured using a validated family health literacy instrument.

Future research should include larger and more diverse samples, combine qualitative and quantitative approaches to further explore the relationships between family health literacy, family functioning, and chronic disease management, and use controlled study designs with longer follow-up periods to evaluate the effectiveness of structured family-centred nursing interventions. Studies incorporating validated measures of family health literacy and longer follow-up periods may contribute to a more comprehensive understanding of how family processes, nursing interventions, and health literacy interact to support sustainable diabetes self-management.

## 5. Conclusions

This embedded mixed-methods multiple-case study suggests that family health literacy and family functioning were closely related to how T2D self-management was understood, negotiated, and incorporated into everyday family life. Across the four family cases, families differed in their ability to interpret and translate diabetes-related information into shared routines, with these differences occurring alongside variations in diabetes self-management. Family communication, mutual support, and the distribution of responsibilities were also associated with how health recommendations were implemented in daily practice, supporting the interpretation of family health literacy as a distributed relational resource rather than exclusively as an individual competence.

The structured application of the MDAIF provided a systematic assessment of family needs, the identification of family nursing diagnoses, and the implementation of tailored interventions adapted to each family’s context. Case-specific changes in family functioning, family nursing diagnoses, communication processes, and selected diabetes self-care behaviours were observed during the period following the tailored interventions. Given the study design, these temporal changes cannot be attributed causally to the interventions. Dietary management, physical activity, and foot care remained the most challenging self-management domains, highlighting the importance of sustained family-centred support.

The findings highlight the potential contribution of Family Health Nursing to chronic disease management in primary healthcare, suggesting that diabetes self-management is related not only to individual knowledge but also to the family’s capacity to communicate, negotiate, mobilise resources, and incorporate health recommendations into everyday routines. Future research should evaluate structured family nursing interventions using controlled study designs, validated measures of family health literacy, longer follow-up periods, more diverse family configurations, and objective clinical outcomes to strengthen the evidence regarding family-centred approaches to diabetes management.

## Figures and Tables

**Table 1:** Demographic, clinical, family, and social characteristics of the participating families.

Family Type	Family 1 Nuclear	Family 2 Extended Family	Family 3 Nuclear Family	Family 4 Nuclear Family
**Household members (*n*)**	2	3	2	2
**Person living with T2D (age/sex)**	73 years, male	68 years, male	66 years, female	66 years, female
**Relationship of participating family members**	Spouse	Spouse and mother	Spouse	Spouse
**Educational level**	4 years of formal education	12 years of formal education	4 years of formal education	Bachelor’s degree
**Employment status**	Retired	Employed	Retired	Retired
**Duration of T2D (years)**	>10 years	>10 years	>10 years	>10 years
**Type of diabetes treatment**	Oral antidiabetic medication	Oral antidiabetic medication and insulin	Oral antidiabetic medication	Oral antidiabetic medication
**Main comorbidities**	Hypertension	Hypertension, dyslipidaemia, heart failure, and chronic bronchitis	Hypertension, osteoarthritis, vertigo syndrome, hypothyroidism, chronic venous insufficiency, and multiple arteriovenous fistulas	Hypertension, osteoporosis, dyslipidaemia, sinusitis, and anxiety
**Presence of diabetes complications**	-	Diabetic foot ulcer (right foot)	-	-
**Primary caregiver/support person**	Spouse	Spouse and mother	Husband	Husband
**Socioeconomic status (Adapted Graffar Scale), score (classification)**	16 (Class III; middle class)	16 (Class III; middle class)	16 (Class III; middle class)	10 (Class II; upper-middle class)
**Family APGAR (Smilkstein), score (interpretation)**	9 (highly functional family)	9 (highly functional family)	10 (highly functional family)	5 (moderate family dysfunction)
**FACES II (cohesion and adaptability), family type**	6 Moderately balanced	4 Intermediate	7 Balanced	7 Balanced
**Holmes and Rahe Social Readjustment Rating Scale, score (interpretation)**	126 (low probability of disease incidence)	142 (low probability of disease incidence)	118 (low probability of disease incidence)	99 (low probability of disease incidence)
**Formal and informal support network**	Primary healthcare unit and extended family	Primary healthcare unit, hospital outpatient diabetic foot clinic, extended family and friends	Primary healthcare unit and extended family	Primary healthcare unit and extended family

**Table 2:** Family nursing intervention process and observed outcomes across the four family cases.

Family	Priority Nursing Diagnosis(es)/Care Focus	Intervention Objective	Tailored Nursing Interventions	Contacts (*n*)	Duration	Outcome Indicator(s)	Observed Outcomes
**Family 1**	T2D self-management behaviours requiring reinforcement	Strengthen T2D self-management through family health literacy and family support.	Promotion of family health literacy; healthy eating and physical activity counselling; medication adherence and foot care promotion; negotiation of self-management goals; reinforcement of family strengths	3	3 months	Diabetes Self-Care Activities Scale	Changes in T2D self-management behaviours and family involvement in diabetes care
**Family 2**	Conjugal satisfaction not maintained (associated with a dysfunctional dynamic relationship); T2D self-management behaviours requiring improvement	Improve conjugal satisfaction and promote family health literacy to strengthen T2D self-management	Promotion of family health literacy; motivational interviewing; family rituals and shared activities; emotional support and communication; lifestyle counselling.	2	3 months	Reassessment of priority family nursing diagnoses and Diabetes Self-Care Activities Scale.	Changes in selected T2D self-management behaviours; no change was observed in the diagnosis of Conjugal satisfaction not maintained
**Family 3**	T2D self-management behaviours requiring reinforcement	Strengthen T2D self-management by building on family strengths and family health literacy	Promotion of family health literacy; reinforcement of healthy eating, physical activity and foot care; therapeutic questioning; reinforcement of family strengths.	3	3 months	Diabetes Self-Care Activities Scale	Maintained effective family functioning and changes in selected T2D self-management behaviours
**Family 4**	Conjugal satisfaction not maintained (associated with a dysfunctional dynamic relationship); dysfunctional family process (associated with ineffective family communication); T2D self-management behaviours requiring reinforcement	Improve family functioning and promote family health literacy to strengthen T2D self-management	Promotion of family health literacy; family rituals; family involvement; communication and assertiveness training; reinforcement of diabetes self-management behaviours.	4	3 months	Reassessment of priority family nursing diagnoses and Diabetes Self-Care Activities Scale.	Changes in conjugal satisfaction and family process, together with changes in T2D self-management behaviours and family support

Tailored family nursing interventions were guided by the MDAIF. Across all cases, systemic family nursing techniques were used according to each family’s needs, including linear, circular, strategic, reflective, and scaling questioning, therapeutic metaphors, therapeutic rituals, positive reinforcement of family strengths, promotion of family health literacy, encouragement of family members to share their individual perspectives, and mutual support among family members. Outcome indicators included the reassessment of priority family nursing diagnoses and diabetes self-management behaviours assessed using the Diabetes Self-Care Activities Scale, according to each family’s assessment and intervention plan.

## Data Availability

The data presented in this study are available from the corresponding author upon reasonable request. The data are not publicly available due to privacy and ethical restrictions.

## Abbreviations

The following abbreviations are used in this manuscript:

APGAR	Adaptation, Partnership, Growth, Affection and Resolve
FACES II	Family Adaptability and Cohesion Evaluation Scales II
MDAIF	Dynamic Model of Family Assessment and Intervention
T2D	Type 2 Diabetes
